# Dasatinib inhibits c-src phosphorylation and prevents the proliferation of Triple-Negative Breast Cancer (TNBC) cells which overexpress Syndecan-Binding Protein (SDCBP)

**DOI:** 10.1371/journal.pone.0171169

**Published:** 2017-01-31

**Authors:** Xiao-Long Qian, Jun Zhang, Pei-Ze Li, Rong-Gang Lang, Wei-Dong Li, Hui Sun, Fang-Fang Liu, Xiao-Jing Guo, Feng Gu, Li Fu

**Affiliations:** 1 Department of Breast Pathology and Lab, Tianjin Medical University Cancer Institute and Hospital, National Clinical Research Center for Cancer, Tianjin, China; 2 Key Laboratory of Cancer Prevention and Therapy, Tianjin, China; 3 Tianjin’s Clinical Research Center for Cancer, Tianjin, China; 4 Key Laboratory of Breast Cancer Prevention and Therapy, Tianjin Medical University, Ministry of Education, Tianjin, China; 5 Department of Breast Surgery, Tianjin Medical University Cancer Institute and Hospital, National Clinical Research Center for Cancer, Tianjin, China; 6 Department of Mathematics, Tianjin Polytechnic University, Tianjin, China; University of South Alabama, UNITED STATES

## Abstract

Triple negative breast cancer (TNBC) progresses rapidly but lacks effective targeted therapies. Our previous study showed that downregulating syndecan-binding protein (SDCBP) in TNBC inhibits the proliferation of TNBC cells. Dasatinib is a new small-molecule inhibitor of c-src phosphorylation. The aim of this study was to investigate if SDCBP is a potential marker to indicate whether a TNBC is suitable for dasatinib therapy. This study applied co-immunoprecipitation to identify the interaction between SDCBP and c-src in TNBC cell lines. In addition, immunohistochemistry was used to investigate SDCBP and tyrosine-419 phosphorylated c-src (p-c-src-Y419) expression in TNBC tissues. SDCBP-overexpressing MDA-MB-231 cells were then constructed to evaluate the effects of dasatinib on SDCBP-induced TNBC progression in vitro and tumor formation in nude mice. We found wild-type SDCBP interacted with c-src and promoted the phosphorylation of c-src; this phosphorylation was completely blocked by dasatinib. SDCBP lacking the PDZ domain had no such effect. Among the 52 consecutive random TNBC cases examined, the expression of SDCBP was consistent with that of p-c-src-Y419, and positively correlated with histological grading or Ki-67 levels. SDCBP overexpression significantly accelerated the proliferation and cell cycle progression of the TNBC cell line MDA-MB-231; these effects were prevented by dasatinib treatment. However, the subsequent inhibition of p27 expression partially restored the proliferation and viability of the TNBC cells. The results of this study suggest that SDCBP interacts with c-src, regulates G1/S in TNBC cells, and enhances tumor cell proliferation by promoting the tyrosine phosphorylation of c-src at residue 419. Dasatinib inhibits such phosphorylation and blocks SDCBP-induced cell cycle progression. Therefore, SDCBP might be an important marker for identifying TNBC cases that are suitable for dasatinib therapy.

## Introduction

Breast cancer is a heterogeneous disease; there are multiple subtypes with different molecular phenotypes, clinical features, and responses to treatment [1]. Classical immunopathological typing is mainly performed based on estrogen receptor alpha (ERα), progesterone receptor (PR), and human epidermal growth factor receptor-2 (HER-2) expression. Triple negative breast cancer (TNBC) refers to breast cancers with negative ERα and PR expression and negative HER-2/Neu receptor overexpression [2]. The mean age of TNBC onset is relatively young and the degree of malignancy is rather high, with a relatively rapid progression, higher local recurrence and distant metastasis rate [3–4], and a lack of specific molecular targets [5]. In recent years, the identification of gene mutations and signaling pathways has led to the discovery of some potential molecular targets, some of which have been used to develop targeted therapies. Although a number of targeted therapeutic drugs for TNBC have been developed, chemotherapy remains as the only clinical option for TNBCs [5]. As such, new therapeutic targets need to be discovered urgently and appropriate therapies need to be developed to overcome the limited treatments for TNBCs.

Syndecan-binding protein (SDCBP), also known as syntenin-1/MDA-9, is a PDZ domain-containing molecule with a large number of interacting ligands [6]. It regulates transmembrane receptors trafficking, tumor cell metastasis, and neuronal-synapse function [7]. Recent studies demonstrated that SDCBP may be an important determinant of malignant phenotypes in many cancers. The effects of SDCBP on melanoma cell malignancy and melanoma metastasis have been investigated extensively [8–11]. Some studies have indicated that SDCBP may also promote the malignant progression of breast cancer [12–14].

Our previous study found a negative correlation between SDCBP and ERα expression while a positive correlation between SDCBP expression and tumor histological grading in breast cancers [15]. SDCBP is overexpressed in multiple TNBC cell lines. Importantly, silencing SDCBP can promote p27 and inhibit cyclin E expression in MDA-MB-231 and BT-549 TNBC cell lines, which blocks the G1/S transition and inhibits cell proliferation [15].

The main translation products of src gene family members are membrane-associated tyrosine protein kinases that lack transmembrane and extracellular domains. They transduce the signals that regulate various cellular processes, including proliferation, mitogenesis, and adhesion [16]. Src family members are usually held in the inactive state and are transiently activated by mitotic events. Numerous human malignancies exhibit increased src expression and activity, suggesting that src may be intimately involved in oncogenesis [17]. c-src non-receptor tyrosine kinase is overexpressed and activated in a large number of human malignancies and has been linked to the development of cancer and progression to distant metastases [18]. Expression of the proto-oncogene c-src is an important cause of spatial and temporal disorder and has abnormal phosphorylation levels in certain tumors [19]. In addition, the binding between focal adhesion kinase and c-src protein plays an important role in tumor metastasis [20, 21]. The tyrosine of c-src at residue 419 is an autophosphorylation site for c-src, which is directly correlated with the level of src TK activity [22].

Morgan et al. [23] showed that elevated src kinase activity attenuates the response to tamoxifen in vitro model and is associated with a poor clinical prognosis of breast cancer. Kanomata et al. [24] showed that 80.9% of breast cancer tissues express tyrosine-phosphorylated c-src. c-src mediates the proliferation of prolactin-dependent T47D and MCF-7 breast cancer cells by activating the FAK/Erk1/2 and PI3K pathways [12]. Functional domain deletion of c-src using point mutations significantly reduces the phosphorylation of FAK (Tyr-925), which is involved in integrin signaling and cell adhesion. It also significantly inhibits the proliferation of MCF-7 cells, resulting in decreased Akt phosphorylation and increased p27 expression [25]. A study by Sanchez-Bailon et al. showed that c-src kinase activity promotes the proliferation, invasion, and metastasis of the TNBC cell line MDA-MB-231 [26]. In melanoma, SDCBP facilitates the formation of active FAK/c-src signaling complexes and the activation of nuclear factor-kappa B (NF-κB) and matrix metalloproteinase (MMP) by directly interacting with c-src to promote tumor invasion and metastasis [10, 27].

On June 28 2006, the U.S. Food and Drug Administration approved dasatinib (Sprycel; Bristol-Myers Squibb), a new small-molecule inhibitor of multiple tyrosine kinases, for the treatment of adults with chronic, accelerated, myeloid, or lymphoid blast phase chronic myeloid leukemia (CML) and Philadelphia chromosome-positive acute lymphoblastic leukemia (Ph (+) ALL) with resistance or intolerance to prior therapy including imatinib [28, 29]. In lung cancer, dasatinib inhibited cell growth by promoting G1/S cell cycle arrest, which was associated with changes in the levels of cyclin D and p27 [30]. Preclinical studies showed single-agent dasatinib inhibited the growth of breast cancer cells with a basal-like phenotype by more frequently than other phenotypes [31, 32]. Preclinical TNBC models also showed that dasatinib exhibited synergistic or additive activity with chemotherapy [33], suggesting that dasatinib may have a clinical benefit in TNBC. Finn et al. conducted a phase II clinical trial to evaluate the efficacy and safety of dasatinib monotherapy in advanced TNBC patients, however, the results indicated that the disease control rate achieved with dasatinib monotherapy was 9.3%, suggesting that the efficacy of dasatinib monotherapy is limited in TNBC patients without individualized screening for molecular markers [34].

To further investigate the effects of SDCBP on TNBC progression and screen for breast cancer with positive treatment outcomes with dasatinib, this study assessed the interaction between SDCBP and c-src in the TNBC cell lines. The consistency of SDCBP and tyrosine phosphorylated c-src expression in TNBC tissues was investigated using immunohistochemistry. Further, a TNBC cell line overexpressing SDCBP was constructed to evaluate the effects of dasatinib on SDCBP-induced breast cancer progression using in vitro experiments and tumor formation experiments in nude mice.

## Materials and methods

### Ethics statement

All human tumor tissues were collected from patients after receiving written consent prior to participation in the study. The protocols for collection and analysis of the samples were approved by the Institutional Review Board of the Tianjin Medical University, in accordance with the current revision of the Helsinki Declaration. The Institutional Animal Care and Use Committee of the Tianjin Medical University approved the use of animal models in this study in accordance with EU Directive 2010/63/EU for animal experiments. All surgery was performed under sodium pentobarbital anesthesia, and all efforts were made to minimize suffering.

### Cell lines

The human TNBC cell lines MDA-MB-231 and BT-549 were purchased from American Type Culture Collection (ATCC^®^ HTB-26^™^ and HTB-122^™^ respectively, Rockville, MD, U.S.A). MDA-MB-231-SDCBP shRNA and BT-549-SDCBP shRNA stable cell lines, with the stable downregulated expression of SDCBP, and the corresponding control cell lines MDA-MB-231-Control shRNA and BT-549-Control shRNA, respectively, were described previously [15].

### P27 siRNA interference

p27 was downregulated by transiently transfecting siRNA; p27 siRNA (sc-29429) and control siRNA (sc-37007) were purchased from Santa Cruz Biotechnology, Inc. (Dallas, TX, U.S.A.). Cells were plated in 35mm culture plates at 60% confluence in normal growth medium. For each transfection, 100 pmol of siRNA oligomer in 250 μl of serum-free cell medium was mixed with 5 μl Lipofectamine 2000 (diluted 1:50) and incubated for 20 min at room temperature. The complex was then added to the cells and incubated cells at 37°C for 48 h before further experiments.

### Western blotting

Total protein from cells was extracted, quantified, and analyzed using western blotting as described previously [15]. Detailed information for antibodies used in this work was listed in S1 Table.

### Establishing MDA-MB-231 cells overexpressing wild-type- or PDZ domain-lacking SDCBP

Total RNA was extracted from MDA-MB-231 cells and used for reverse transcription. The products of reverse transcription were used for PCR using the primers listed in S2 Table to yield wild-type SDCBP or SDCBP lacking the PDZ domain. The amplified fragments were individually digested with BamH1 and then cloned into BamH1 and EcoRV restriction sites in the eukaryotic pCMV-Tag2B expression vector (Stratagene, La Jolla, CA, U.S.A) to yield constructs containing the human wild-type SDCBP coding region and SDCBP lacking the PDZ domain in-frame with FLAG. DNA sequencing was performed to verify that the inserted sequence was correct. Transient transfections were performed in 293T cells. SDCBP and FLAG tag-specific antibodies were used to verify that both forms of SDCBP were expressed normally using western blotting. The verified vectors were named pCMV-Tag2B (SDCBP) and pCMV-Tag2B (SDCBP-PDZ), respectively.

MDA-MB-231 cells were transfected with pCMV-Tag2B (SDCBP) or pCMV-Tag2B (SDCBP-PDZ) using Lipofectamine 2000 (Life Technologies Corporation, Grand Island, NY, U.S.A) according to the manufacturer’s protocol; empty vector was used as a control. The corresponding exogenous protein overexpression was evaluated using western blotting after the cells were cultured for 5 weeks in the appropriate medium containing 0.5 mg/ml of G418 (Sigma-Aldrich, St. Louis, MO, U.S.A).

### Co-immunoprecipitation to study protein-protein interactions

For co-immunoprecipitations, one milliliter of cell lysates was incubated overnight at 4°C with antibodies against c-src or SDCBP coupled to protein G Sepharose. Immunoprecipitates were extensively washed, and the eluted precipitates were resolved by SDS-PAGE, transferred, and probed with the appropriate antibodies according to a previous literature [10].

### Sampling and immunohistochemistry

TNBC samples were collected from consecutive breast cancer cases from January 2014 to February 2014, as described in our previous study [35]. The immunohistochemistry experiment was conduced in December 2014. In order to protect the patient's privacy, we removed the names of the patients, their hospital numbers, the pathological numbers of the specimens, and so on, during data collection. ERα and PR staining was performed and scored in accordance with the methods described previously [15]. Cases were considered negative for ERα and PR if nuclear staining was present with a final score <50 [36]. Her-2 immunohistochemistry and fluorescence in situ hybridization were performed according to the ASCO/CAP 2013 guidelines. Fifty-two TNBC cases were collected, including 3 cases that were ERα and PR negative, 2+ in Her-2 immunohistochemistry, and with equivocal value for Her-2 dual-probe FISH). Staining for p-c-src-Y419 and SDCBP were performed as reported previously [15]. The p-c-src-Y419 and SDCBP statuses were determined by scoring the proportion of stained cells and measuring the intensity of the cytoplasmic staining [15]. This scoring system gives a final score ranging from 0 to 300, and the final score was classified as follows: 0–50, negative; 51–100, weakly positive; 101–200, moderately positive; and 201–300, strongly positive. All cases were evaluated by two pathologists independently and any discrepancy was resolved by a group discussion.

### Cell growth curve and cell cycle analyses

MDA-MB-231-SDCBP and MDA-MB-231-Neo cells were used for experiments and the methods were identical to those reported previously [15].

### Dasatinib treatment in MDA-MB-231 cells

Dasatinib (10 mM) dissolved in DMSO [37] was purchased from ApexBio Technology (Houston, TX, U.S.A, BMS-354825). The final concentration of dasatinib for the treatment of MDA-MB-231 cells was 100 nM. Cell cycle analysis and viability tests were performed in different groups of cells after 48-h treatment.

### MDA-MB-231 cell viability analysis

Trypan blue staining was used to assess the viability of cells as described in a previous study [38]. SDCBP overexpressing, control MDA-MB-231, or MDA-MB-231 cells transfected with p27 siRNA with 70% confluency were cultured in serum-free RPMI-1640 medium and treated with dasatinib for 48 h. They were then stained using a Trypan Blue Staining Kit (Beyotime Institute of Biotechnology, Shanghai, China) according to the manufacturer’s instructions to measure cell viability. The average viability of control MDA-MB-231 cells (neither overexpressing SDCBP nor treated with dasatinib) from triplicate experiments was defined as “1” to calculate the relative cell viability of the different groups of cells.

### In vivo tumorigenicity

MDA-MB-231-SDCBP and MDA-MB-231-Neo cells were inoculated into nude mice to create a xenograft tumor model as described previously [15]. Nude mice were randomly divided into the following four groups (six animals per group): non-overexpressing SDCBP group (MDA-MB-231-Neo inoculated nude-mice without dasatinib administration); non-overexpressing SDCBP, dasatinib-treated group (MDA-MB-231-Neo inoculated nude-mice with dasatinib administration); SDCBP overexpressing without dasatinib treatment group (MDA-MB-231-SDCBP inoculated nude-mice without dasatinib administration); and SDCBP overexpressing, dasatinib-treated group (MDA-MB-231-SDCBP inoculated nude-mice with dasatinib administration). A total of 1 × 10^6^ cells were inoculated into each nude mouse. To prepare the working solution for the oral gavage administration, dasatinib was dissolved in a 50:50 mixture of polypropylene glycol (Sigma-Aldrich, St. Louis, MO, U.S.A) and water. Animals were treated with dasatinib every other day from day 7 to day 21 after tumor cell inoculation for a total of eight administrations (10 mg/kg q.o.d.). Animals in the control group were treated with the same volume of the above solution without dasatinib for the same duration as the experimental group. At the end of the seventh week afeter inoculation, the tumors were removed at necropsy, post-fixed in 4% neutral buffered formalin for 24 h, paraffin-embedded, and serially sectioned into 4-μm thick sections. Ki-67 rabbit polyclonal antibody (sc-15402, Santa Cruz Biotechnology, Inc., Dallas, TX, U.S.A.) was used for Ki-67 immunohistochemical staining on the tissue sections, and the Ki-67 index was calculated as described previously [39].

### Statistical analyses

The SPSS 15.0 software package (SPSS, Inc., Chicago, IL, U.S.A) was used for statistical analysis. The results were analyzed using t-tests for normally distributed data. The correlations between SDCBP staining and other clinical pathological features were analyzed using non-parametric spearman correlation tests. Mann-Whitney U tests were used to determine the significance of the differences between two groups of data with a skewed distribution. A two-sided P value <0.05 was considered statistically significant in all analyses.

## Results

### Selection of stable MDA-MB-231 cell clones over-expressing wild-type- or PDZ domain-lacking SDCBP

**Fig 1:**
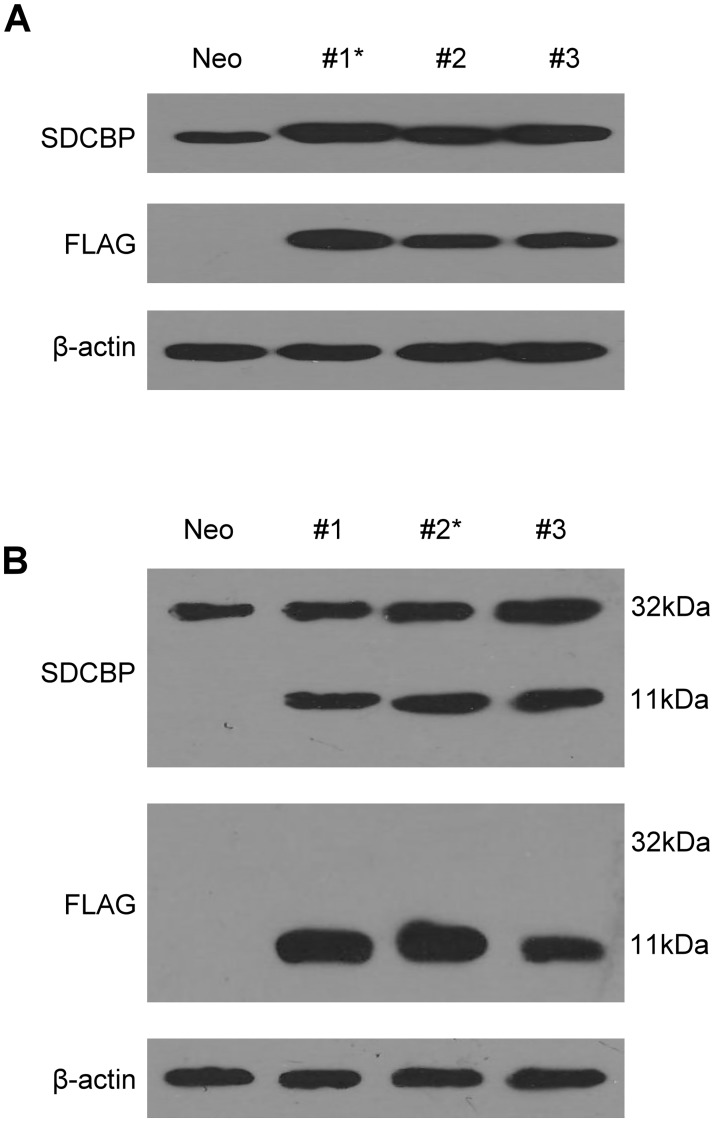
Screening MDA-MB-231 cells stably overexpressing wild-type SDCBP (A) and SDCBP with the PDZ domains deleted (B). * Represents the clones selected for subsequent studies.

One stable clone with the highest wide-type SDCBP overexpression and one stable clone with the highest overexpression of SDCBP lacking the PDZ domain were identified and named MDA-MB-231-SDCBP and MDA-MB-231-SDCBP-PDZ, respectively (Fig 1 and S1 Fig). The control clone stably transfected with empty pCMV-Tag2B vector was named MDA-MB-231-Neo.

### The interaction between SDCBP and c-src

**Fig 2:**
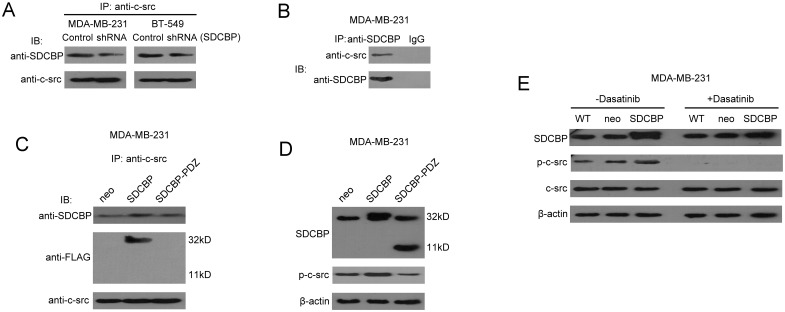
Interaction between SDCBP and c-src in TNBC cell lines. (A) The c-src-specific antibody was used to immunoprecipitate from the cell lyastes of MDA-MB-231-SDCBP shRNA or BT-549 SDCBP shRNA and from the cell lysates of their corresponding control shRNA cells; the precipitated product were then analyzed using the SDCBP-specific antibody. (B) The SDCBP-specific antibody (or IgG as a control) was used to immunoprecipitate from MDA-MB-231 cell lysates; samples were then analyzed using the specific c-src antibody. (C) The c-src-specific antibody was used to immunoprecipitate wild-type SDCBP and SDCBP lacking the PDZ domains from their corresponding stably transfected MDA-MB-231 cell lysates. The precipitated products were then analyzed using the SDCBP-specific and FLAG antibodies. (D) Immunoblotting was used to evaluate the effects of SDCBP or its mutant protein lacking the PDZ domains overexpression on the tyrosine phosphorylation of c-src at residue 419. (E) Immunoblotting was used to evaluate the effects of SDCBP overexpression on c-src and p-c-src-Y419 levels in the presence or absence of 100 nM dasatinib treatment. WT represents wild-type MDA-MB-231 cells.

A specific c-src antibody was used to perform co-precipitations in MDA-MB-231-SDCBP shRNA and BT-549-SDCBP shRNA cell lysates or in their corresponding control shRNA stable-transfected cells’ lysates. Co-precipitated SDCBP protein levels were reduced significantly in the SDCBP shRNA cells (Fig 2A and S2 Fig). Similarly, co-precipitations performed in MDA-MB-231 cell lysates using the SDCBP-specific antibody could pull-down c-src protein; control immunoprecipitations performed using IgG did not bind to SDCBP and precipitate c-src protein (Fig 2B and S2 Fig).

MDA-MA-231-SDCBP, MDA-MB-231-SDCBP-PDZ and MDA-MD-231-Neo cells were used in co-precipitation experiments using the specific c-src antibody. Immunoblotting confirmed that co-precipitated SDCBP was significantly up-regulated in the MDA-MB-231-SDCBP group. Immunoblots using the FLAG-tag antibody revealed a specific 32-kD protein band in the MDA-MB-231-SDCBP group; however, a specific band corresponding to the molecular weight of SDCBP with two missing PDZ domains was not observed in the MDA-MB-231-SDCBP-PDZ group (Fig 2C and S2 Fig), suggesting that c-src precipitated wild-type SDCBP but not SDCBP lacking the PDZ domains.

We also found p-c-src-Y419 expression was significantly higher in the MDA-MB-231-SDCBP group compared with the MDA-MB-231-Neo group. However, there was no difference in p-c-src-Y419 levels between the MDA-MB-231-SDCBP-PDZ and MDA-MB-231-Neo groups (Fig 2D and S2 Fig). These results suggest that widetype SDCBP and c-src physically interact with each other at the PDZ domain of SDCBP. MDA-MB-231-Neo, MDA-MD-231-SDCBP, and untransfected MDA-MB-231 cells were incubated with normal culture medium containing 100 nM dasatinib for 48 h. As a control, the three groups of cells were also incubated with normal culture medium containing the same volume of DMSO for 48 h. In the control group, SDCBP overexpression caused an increase in p-c-src-Y419 expression. However, p-c-src-Y419 expression was nearly undetectable in dasatinib-treated cells, and SDCBP overexpression did not restore p-c-src-Y419 expression (Fig 2E and S2 Fig).

### The expression of SDCBP and p-c-src-Y419 in breast cancer tissues

**Fig 3:**
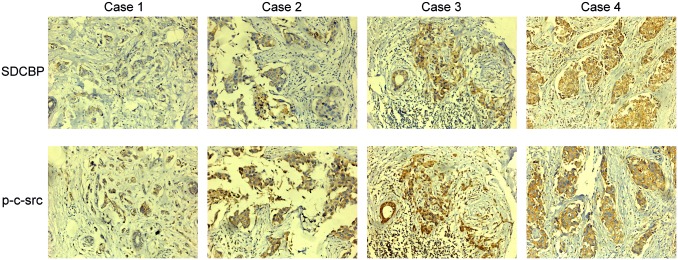
The expression of p-c-src-Y419 and SDCBP in triple-negative breast cancer tissue (×200). Case 1 has weak p-c-src-Y419 and negative SDCBP expression, Case 2 has weak p-c-src-Y419 and weak SDCBP expression, Case 3 has moderate p-c-src Y419 and moderate SDCBP expression, and Case 4 has strong p-c-src-Y419 and strong SDCBP expression.

Immunohistochemical staining, analysis, and grading of SDCBP and p-c-src-Y419 expression in 52 consecutive TNBC specimens (S3 Table) showed that SDCBP and p-c-src-Y419 were both mainly localized in the cytoplasm (Fig 3). None of the 52 consecutive TNBC cases were p-c-src-Y419 negative. Non-parametric Spearman’s correlation tests showed significant correlations between SDCBP expression and histological tumor grading (P = 0.001), SDCBP expression and Ki-67 index (P = 0.012), and SDCBP expression and p-c-src-Y419 expression (P = 0.009). However, a significant association between SDCBP expression and tumor TNM staging or lymph node status was not positively established (P = 0.096 and P = 0.101, respectively; Table 1).

**Table 1:** Syndecan-binding protein (SDCBP) and tyrosine-419 phosphorylated c-src (p-c-src-Y419) expression and the pathological features of triple-negative breast cancers.

Pathological features	Cases	SDCBP*(%)				rs	P value
		Negative	Weak	Moderate	Strong		
Histological grading						0.442	0.001
I	3	2 (66.7)	1 (33.3)	0 (0.0)	0 (0.0)		
II	22	6 (27.3)	5 (22.7)	6 (27.3)	5 (22.7)		
III	27	1 (3.7)	1 (3.7)	15 (55.6)	10 (37.0)		
Lymph node status						0.230	0.101
N0	21	3 (14.3)	6 (28.6)	6 (28.6)	6 (28.6)		
N1	10	3 (30.0)	0 (0.0)	5 (50.0)	2 (20.0)		
N2	13	3 (23.1)	1 (7.7)	5 (38.5)	4 (30.8)		
N3	8	0 (0.0)	0 (0.0)	5 (62.5)	3 (37.5)		
pTNM staging						0.233	0.096
I	13	2 (15.4)	5 (38.5)	2 (15.4)	4 (30.8)		
II	17	4 (23.5)	1 (5.9)	9 (52.9)	3 (17.6)		
III-IV	22	3 (13.6)	1 (4.5)	10 (45.5)	8 (36.4)		
Ki-67 index (%)	52	25.0±14.8	46.4± 29.0	61.2± 28.0	63.0±20.3	0.345	0.012
p-c-src-Y419*						0.360	0.009
Negative	0	0 (0.0)	0 (0.0)	0 (0.0)	0 (0.0)		
Weak	8	3 (37.5)	1 (12.5)	3 (37.5)	1 (12.5)		
Moderate	23	5 (21.7)	4 (17.4)	7 (30.4)	7 (30.4)		
Strong	21	1 (4.8)	2 (9.5)	11 (52.4)	7 (33.3)		

* The staining score (range, 0–300) obtained from SDCBP and p-c-src-Y419 immunohistochemistry were directly used for non-parametric Spearman’s correlation tests.

### Effects and mechanisms of SDCBP over-expression and dasatinib treatment on TNBC progression

**Fig 4:**
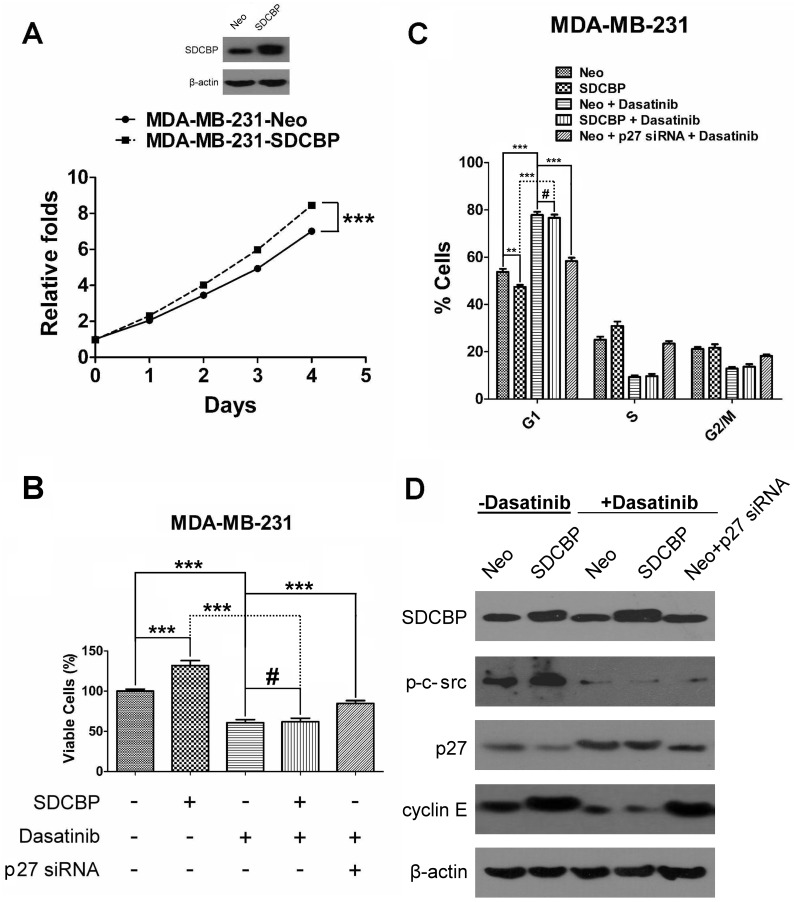
The effects of SDCBP overexpression and dasatinib treatment on cell proliferation, viability, the cell cycle, and its important regulatory molecules in MDA-MB-231 cells. (A) MTT assays were used to evaluate the effects of SDCBP overexpression on the proliferation of MDA-MD-231 cells. (B) Trypan blue staining was used to evaluate the effects of SDCBP overexpression and/or 100 nM dasatinib administration, as well as 100 nM dasatinib together with p27 siRNA on cell viability (#P > 0.05; ***P < 0.001). (C) Flow cytometry was used to evaluate the effects of SDCBP overexpression and/or 100 nM dasatinib administration, as well as 100 nM dasatinib together with p27 siRNA on the cell cycle (#P > 0.05; ***P < 0.001). (D) Immunoblotting was used to evaluate the effects of SDCBP overexpression and/or 100 nM dasatinib, as well as 100 nM dasatinib together with p27 siRNA on the expression of p-c-src-Y419, p27, and cyclin E.

Growth curves revealed that SDCBP overexpression accelerated the growth of MDA-MB-231 cells. At the end of day 1, 2, 3 and 4, the A490 ratio (calculated as our previous study[15]) of MDA-MB-231-SDCBP cells was increased significantly compared with MDA-MB-231-Neo cells (P = 0.016, P < 0.001, P < 0.001, and P < 0.001, respectively; Fig 4A).

Then, the viability of the five groups of MDA-MB-231 cells (i.e., non-overexpressing SDCBP group; non-overexpressing SDCBP, dasatinib-treated group; SDCBP overexpressing without dasatinib treatment group; SDCBP overexpressing, dasatinib-treated group; and SDCBP non-overexpressing, dasatinib and p27 siRNA-treated group) was assessed. SDCBP overexpression significantly enhanced the viability of cells without dasatinib treatment (P < 0.001), whereas the viability of dasatinib-treated MDA-MB-231-Neo or MDA-MB-231-SDCBP cells was significantly lower than that of their corresponding untreated ones (P < 0.001 and P < 0.001, respectively). Although SDCBP overexpression did not restore the low viability (P = 0.623), treatment with p27 siRNA partially restored cell viability (P < 0.001, Fig 4B).

Next, the cell cycle was analyzed in the above-mentioned five groups and the proportion of cells in G1 phase was compared. SDCBP overexpression significantly decreased the proportion of MDA-MB-231 cells in G1 phase (P = 0.002). Dasatinib treatment significantly increased the number of MDA-MB-231-Neo or MDA-MB-231-SDCBP cells in G1 phase (P < 0.001 and P < 0.001, respectively). Although this could not be significantly reduced by SDCBP overexpression (P = 0.343), the application of p27 siRNA reduced the number of cells in G1 phase (P < 0.001, Fig 4C).

The protein expression of p-c-src-Y419, cyclin-dependent kinases inhibitor p27 and the G1/S-checkpoint-related cyclin E was analyzed in the five groups of cells using immunoblotting. In MDA-MB-231 cells not treated with dasatinib, p-c-src-Y419 and cyclin E were upregulated, whereas p27 was downregulated when SDCBP was overexpressed. In cells treated with dasatinib, only trace amounts of p-c-src-Y419 were expressed and cyclin E expression was significantly reduced; however, p27 expression was significantly increased. Under such conditions, SDCBP overexpression could not restore the expression of the above three proteins. Nevertheless, p27 siRNA partly recovered cyclin E expression but not p-c-src-Y419 expression (Fig 4D and S3 Fig).

### Effects of dasatinib and SDCBP on MDA-MB-231 cell tumorigenicity in nude mice

As described in the Methods section, nude mice were divided into different group and treated with dasatinib. Tumor volumes were measured once a week from the first week after tumor cell inoculation. All mice developed tumors.

**Fig 5:**
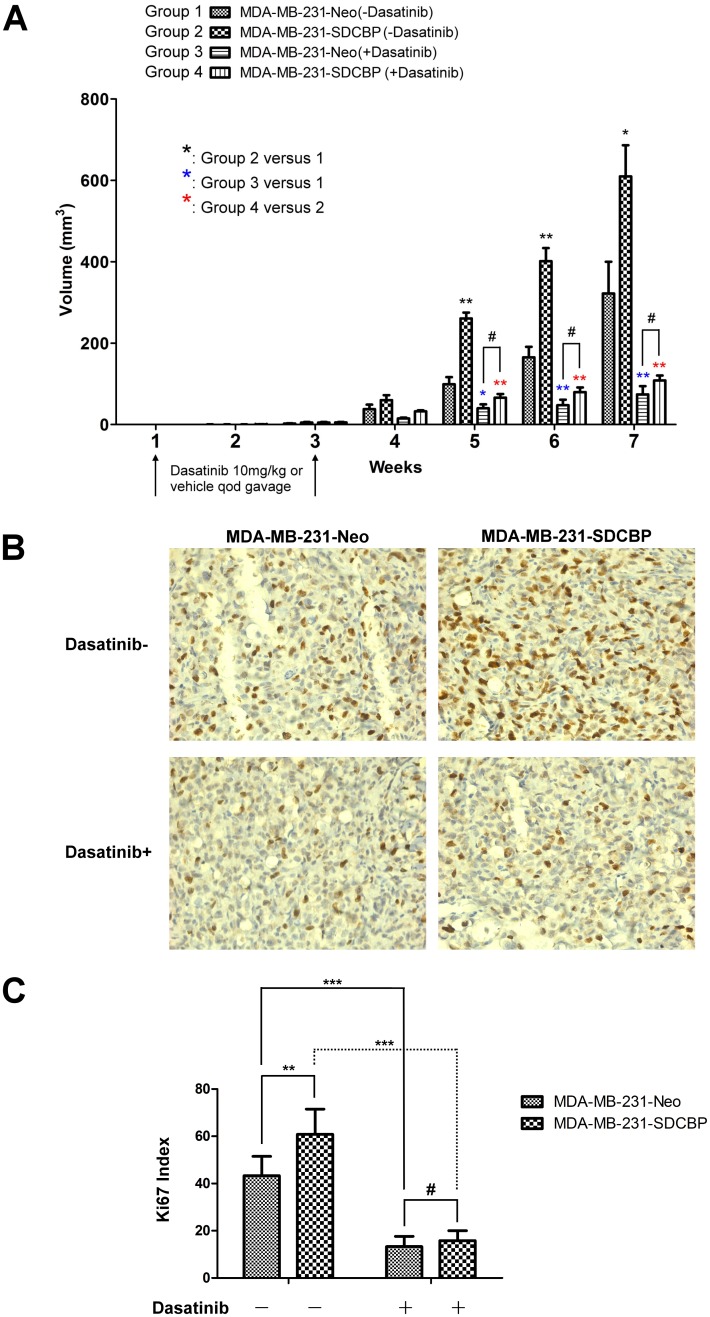
The effects of SDCBP overexpression and the intragastric administration of dasatinib on tumor growth in nude mice inoculated with MDA-MB-231 cells. (A) Comparison of the tumor volumes at the end of each week after inoculation in different groups of nude mice inoculated with MDA-MB-231 cells that received SDCBP overexpression and/or the intragastric administration of dasatinib (#P > 0.05; *P < 0.05; **P < 0.01). (B) Representative immunohistochemical-staining images of Ki-67 expression in tumors extracted from different groups of nude mice inoculated with MDA-MB-231 cells that received SDCBP overexpression and/or the intragastric administration of dasatinib (× 200). (C) Differences in the Ki-67 index among groups (#P > 0.05; **P < 0.01; ***P < 0.001).

Dasatinib administration significantly inhibited tumor growth in nude mice inoculated with MDA-MB-231 cells, regardless of the presence of SDCBP overexpression. In two dasatinib-treated groups, the tumor volumes were both significantly smaller than their corresponding dasatinib untreated groups starting from the end of week 5 (for MDA-MB-231-Neo inoculated tumors, P = 0.015, P = 0.002, and P = 0.002 at the end of week 5, 6, and 7, respectively; for MDA-MB-231-SDCBP inoculated tumors, P = 0.002, P = 0.002, and P = 0.002 at the end of week 5, 6, and 7, respectively, Fig 5A). At the end of the seventh week after inoculation, dasatinib gavage reduced the volume of the tumors extracted from nude mice inoculated with MDA-MB-231-Neo and MDA-MB-231-SDCBP cells by 76.72% and 82.25%, respectively, compared with their corresponding control groups. In dasatinib untreated groups, the volumes of MDA-MB-231-SDCBP tumors were significantly larger than that of MDA-MB-231-Neo tumors at the end of week 5, 6, and 7 (P = 0.002, P = 0.004, and P = 0.041, respectively, Fig 5A). At the end of the seventh week after inoculation, the median volume of the MDA-MB-231-SDCBP tumors was 91.85% larger than that of the MDA-MB-231-Neo tumors. However, in two dasatinib-treated groups there was no significant difference in tumor volume at the end of week 5, 6, and 7 (P = 0.065, P = 0.093, and P = 0.310, respectively, Fig 5A).

Ki-67 staining of tumor tissues (Fig 5B) revealed that the intragastric administration of dasatinib significantly inhibited Ki-67 expression in both MDA-MD-231-Neo (P < 0.001) and MDA-MB-231-SDCBP (P < 0.001) cells. In dasatinib untreated groups, tumors formed from MDA-MB-231-Neo cells had a remarkably lower Ki-67 index than MDA-MB-231-SDCBP tumors (P = 0.010). However, in two dasatinib-treated groups, there were no significant differences in Ki-67 index (P = 0.332, Fig 5C).

## Discussion

The outcome of TNBC patients has significantly improved with adjuvant chemotherapy, which reduces the risk of death by ~50%. However, there has been limited progress in incorporating additional systemic therapies into the management of TNBCs [40, 41] and the improvements in survival have plateaued [42]. In addition, TNBC patients are currently the subgroup of breat cancer patients with the worst outcome [43, 44].

Our previous study demonstrated that SDCBP plays an important role in TNBC proliferation [15]. A study by Sanchez-Bailon et al. showed that c-src kinase activity promotes the proliferation, invasion, and migration of MDA-MB-231 TNBC cells [26]. Dasatinib, an oral small molecular inhibitor of both src and abl kinases, significantly inhibits the tyrosine phosphorylation of c-src at residue 419. The current study combined TNBC, SDCBP, and c-src to achieve the following research progress.

Co-immunoprecipitation is an effective method to identify physical interactions between two proteins in intact cells. By constructing cell lines overexpressing wild-type SDCBP and SDCBP lacking the PDZ domains and using co-immunoprecipitation, we identified a physical interaction between wild-type SDCBP and c-src in TNBC cells, even though the interaction may be direct or indirect. The PDZ domain is the key domain for this interaction. In addition, only wild-type SDCBP could promote the tyrosine phosphorylation of c-src at residue 419. Treating cells with 100 nM dasatinib completely inhibited the tyrosine phosphorylation of c-src at residue 419 in MDA-MB-231 cells, which could not be restored by overexpressing SDCBP. This experiment suggests that SDCBP interacts with c-src and promotes the tyrosine phosphorylation of c-src at residue 419; this was completely blocked by dasatinib.

The analysis of 52 consecutive cases of TNBC in our hospital from January 2014 to February 2014 showed that the expression level of SDCBP was consistent with the extent of p-c-src-Y419. In addition, SDCBP expression was positively correlated with histological grading and Ki-67 index. These results suggest that the interaction between SDCBP and c-src promotes tyrosine phosphorylation of c-src at the 419 both in vitro experiments and in cancer tissues in TNBC.

c-src plays an important role in cell cycle progression in many tumor cells, and suppressing c-src downregulates cyclin D1 and cyclin E and upregulates p27 Kip1 [45]. The kinase inhibitor p27 Kip1 regulates G1 phase of the cell cycle. Src inhibitors increase cellular p27 stability, whereas Src overexpression accelerates p27 proteolysis [46]. Cyclin E, the late G1 cyclin that together with its catalytic subunit Cdk2 regulates the transition from G1 to S phase, is downregulated by c-src suppression [45, 46]. High levels of cyclin E correlate strongly with a poor outcome in patients with breast cancer [47]. The overexpression of p27 can also reduce cyclin E levels [48]. Our previous study demonstrated that SDCBP is overexpressed in TNBC, which accelerates the transition of tumor cells through the G1/S checkpoint by increasing cyclin E expression to promote the proliferation of TNBC cells [15]. To further assess if the interaction between SDCBP and c-src promoted the proliferation of TNBC cells and to investigate the effect of dasatinib, we analyzed the effects of SDCBP overexpression and/or dasatinib treatment and p27 downregulation on the cell cycle and viability in MDA-MB-231 cells; we also investigated changes in the levels of p-c-src-Y419, the cell cycle regulatory molecule p27, and cyclin E. The results showed that the overexpression of wild-type SDCBP significantly accelerated the cell proliferation and viability of MDA-MB-231 TNBC cells. However, this acceleration of cell proliferation and viability was inhibited by dasatinib treatment. Subsequently, interfering with p27 expression reduced the number of cells arrested in G1 phase under dasatinib treatment. Immunoblotting revealed that the overexpression of wild-type SDCBP promoted the tyrosine phosphorylation of c-src at residue 419, downregulated p27 expression, and upregulated cyclin E expression. Conversely, dasatinib treatment reduced p-c-src-Y419 expression to trace levels, and the above-mentioned effects of SDCBP overexpression were also lost. Although the application of siRNA to downregulate p27 expression did not increase p-c-src-Y419 expression, it partially restored cyclin E expression. These findings suggest that SDCBP promoted TNBC cell proliferation and enhanced cell viability by enhancing the tyrosine phosphorylation of c-src at residue 419. These two effects might be achieved by inhibiting p27 expression via p-c-src-Y419 to further increase cyclin E expression and promote G1/S cell cycle progression. Dasatinib treatment might inhibit the cell proliferation and cell viability of TNBC by inhibiting the tyrosine phosphorylation of c-src at residue 419, and thus the effect of SDCBP overexpression was blocked. The application of siRNA to interfere p27 expression partially recovered cell cycle progression, suggesting that p27 may be located downstream of p-c-src-Y419 in this signal transduction pathway. Thus, inhibiting p27 expression partially restored cell cycle progression by increasing cyclin E expression.

A nude mice tumor xenograft model showed that SDCBP promoted MDA-MB-231 tumor growth, which was inhibited by the gavage administration of dasatinib. No significant differences in tumor volumes were found between MDA-MB-231-SDCBP and MDA-MB-231-Neo tumors at different time points when dasatinib was administrated. Indeed, MDA-MB-231-SDCBP inoculated nude mice lost more proportion of tumor volumes under dasatinib administration than MDA-MB-231-Neo inoculated nude mice did (82.3% versus 76.7%). Ki-67 (also known as MKI67) is a cellular marker for proliferation [49]. Measurement of the Ki-67 indices in MDA-MB-231-SDCBP and MDA-MB-231-Neo tumors revealed results that were consistent with the tumor growth findings. Although the overexpression of SDCBP significantly elevated the Ki-67 indices of the inoculated tumors, dasatinib significantly reduced the Ki-67 indices, which could not be restored by overexpressing SDCBP. Therefore, the results of the nude mice tumor xenografts further confirmed that dasatinib blocks SDCBP overexpression-induced TNBC tumor growth, suggesting that dasatinib might be a targeted therapy for TNBCs that overexpress SDCBP.

This study revealed a signal transduction pathway commonly found in TNBC: the interaction between SDCBP and c-src promotes the tyrosine phosphorylation of c-src at residue 419, which facilitates the transition of cells through the G1/S checkpoint to promote tumor cell proliferation. Dasatinib inhibited the tyrosine phosphorylation of c-src at residue 419 and blocked the ability of SDCBP to promote cell cycle progression. Finn et al. conducted a phase II clinical trial to assess the efficacy and safety of dasatinib monotherapy in advanced TNBC patients. Their results revealed that the disease control rate was 9.3%, suggesting that the efficacy of dasatinib monotherapy was limited in TNBC patients without individual screening for molecular markers [34]. We speculate that SDCBP might be an important marker for identifying TNBC likely to responds to dasatinib therapy. SDCBP may be even a better marker for dasatinib sensitivity than phospho-Y419 of src, because: Dasatinib can totally block the effect of SDCBP on the proliferation of TNBC cells. Furthermore, the positive rate of p-c-src-Y419 in TNBC tissue is extremely high (100% in 52 consecutive TNBC specimens), and the percentage of moderate and strong immunohistochemical staining is as high as 84.6%, which may not be benefit for individual screening. Also, the interaction between SDCBP and other src-family kinases, which may also promote the progression of TNBC and be a target of dasatinib, cannot be totally excluded. SDCBP has three different isoforms, although the differences in the sequences and molecular weights of them are very small. There are about 10 amino acids inconsistency near the N-terminus between isoforms 1 and 2 [50]. The different functions of these SDCBP isoforms are worth further study.

In TNBC, identifying signal molecules upstream of SDCBP would be worthwhile. In addition, since SDCBP is not a kinase nor has any kinase-like domains, it would be useful to know whether other signaling molecules are involved in the interaction between SDCBP and c-src, and further phosphorylates c-src at Y419. A large number of animal experiments and clinical trials will be needed to assess whether SDCBP could be used as a marker for identifying TNBC patients likely to respond to dasatinib therapy.

## Supporting information

S1 Table Detailed information for antibodies used in this work. (DOC)

S2 Table Primers used to generate the eukaryotic expression vectors over-expressed wide-type or PDZ domain-deleted SDCBP. (DOC)

S3 Table Detailed features of 52 consecutive cases of triple-negative breast cancer from January to February 2014. (XLS)

S1 Fig Original information for Fig 1(western blot). (JPG)

S2 Fig Original information for Fig 2(western blot). (JPG)

S3 Fig Original information for Fig 4D(western blot). (JPG)
