# Supplementary material for: Dasatinib inhibits c-src phosphorylation and prevents the proliferation of Triple-Negative Breast Cancer (TNBC) cells which overexpress Syndecan-Binding Protein (SDCBP)
Source: PLoS One. 2017 Jan 31;12(1):e0171169. doi: 10.1371/journal.pone.0171169 (PMC5283743; doi:10.1371/journal.pone.0171169)
Supplement: S1 Table — (DOC) [file pone.0171169.s001.doc]

**S1 Table Detailed information for antibodies used in this work.**

| **Name** | **Type** | **Supplier** | **Catalog #** | **Origin** | **Dilution Ratio** | |
| --- | --- | --- | --- | --- | --- | --- |
|  |  |  |  |  | **For WB*** | **For IHC**** |
| cyclin E Antibody (M-20) | rabbit polyclonal | Santa Cruz Biotechnology, Inc. | sc-481 | Dallas, TX, U.S.A. | 1:1000 |  |
| β-Actin Antibody (C4) | mouse monoclonal | Santa Cruz Biotechnology, Inc. | sc-47778 | Dallas, TX, U.S.A. | 1:1500 |  |
| p27 Kip1 (D69C12) | Rabbit monoclonal | Cell Signaling Technology, Inc. | #3686 | Danvers, MA, U.S.A | 1:1000 |  |
| c-Src Antibody (B-12) | mouse monoclonal | Santa Cruz Biotechnology, Inc. | sc-8056 | Dallas, TX, U.S.A | 1:500 |  |
| syntenin-1 Antibody (N-20) | goat polyclonal | Santa Cruz Biotechnology, Inc. | sc-19379 | Dallas, TX, U.S.A | 1:500 | 1:75 |
| p-c-Src Antibody (Tyr 419) | Rabbit polyclonal | Santa Cruz Biotechnology, Inc. | sc-101802 | Dallas, TX, U.S.A | 1:500 | 1:75 |

***, Westernblot**

****, Immunohistochemistry**
