# Supplementary material for: Dasatinib inhibits c-src phosphorylation and prevents the proliferation of Triple-Negative Breast Cancer (TNBC) cells which overexpress Syndecan-Binding Protein (SDCBP)
Source: PLoS One. 2017 Jan 31;12(1):e0171169. doi: 10.1371/journal.pone.0171169 (PMC5283743; doi:10.1371/journal.pone.0171169)
Supplement: S2 Table — (DOC) [file pone.0171169.s002.doc]

**S2 Table Primers used to generate the eukaryotic expression vectors over-expressed wide-type or PDZ domain-deleted SDCBP.**

| **.** | **Amplified Fragment Length (bp)** | **Annealing Temperature (℃)** | **Primer Name** | **Sequence (from 5’ to 3’)** |
| --- | --- | --- | --- | --- |
| SDCBP | 950 | 55 | SDCBP-FOR -BAMH I | CGGGATCCATGTCTCTCTATCCATCTCTCGAAG |
|  |  |  | SDCBP-REV | GCCAAAGAAGGAAACTGGAGAC |
| SDCBP-PDZ | 342 | 55 | SDCBP-FOR -BAMH I | CGGGATCCATGTCTCTCTATCCATCTCTCGAAG |
|  |  |  | SDCBP-PDZ -REV | TTAACGAATCCCTTGCTTAATTTC |
