# Supplementary figures and images for: Dasatinib inhibits c-src phosphorylation and prevents the proliferation of Triple-Negative Breast Cancer (TNBC) cells which overexpress Syndecan-Binding Protein (SDCBP)
Source: PLoS One. 2017 Jan 31;12(1):e0171169. doi: 10.1371/journal.pone.0171169 (PMC5283743; doi:10.1371/journal.pone.0171169)

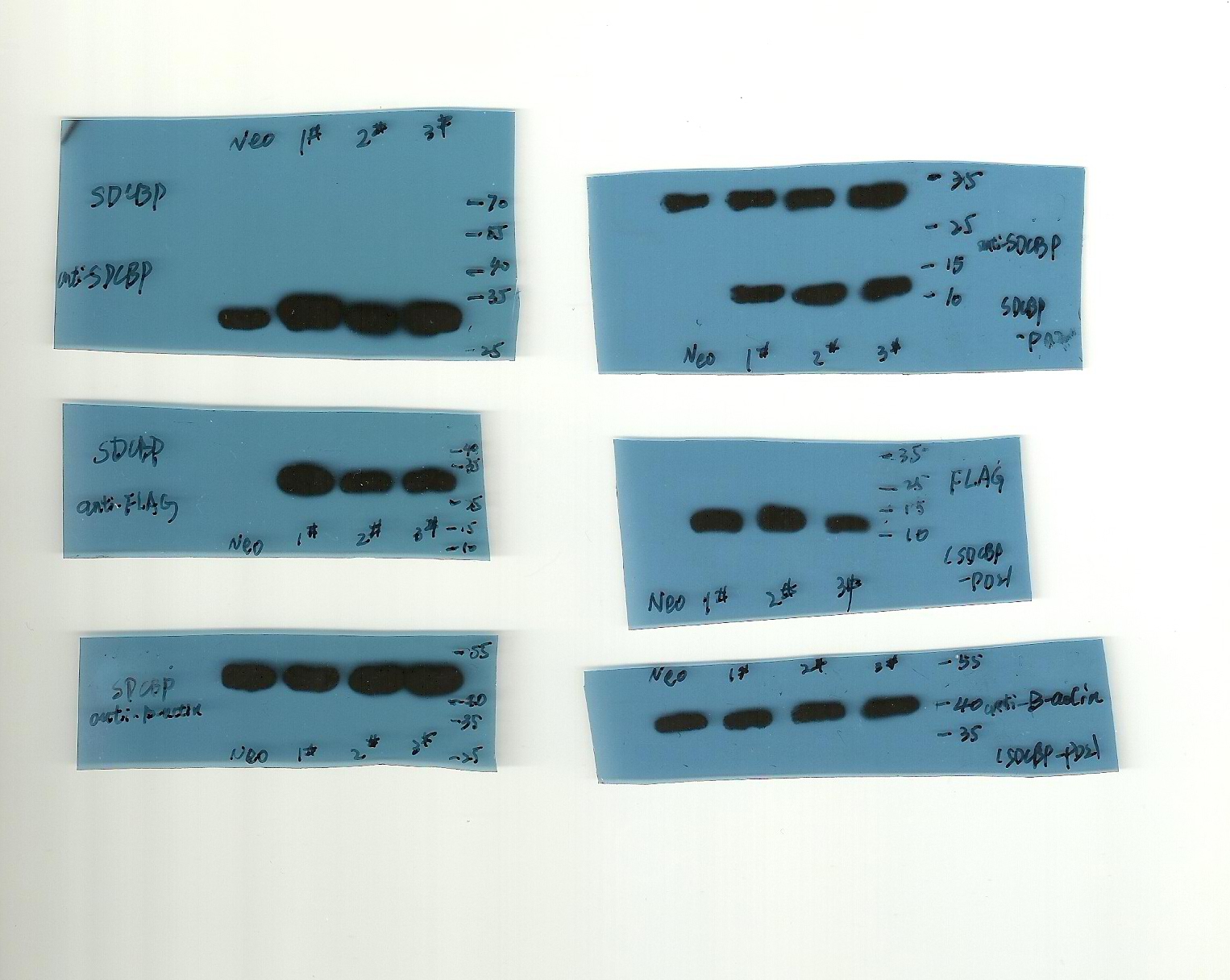

Supplement: S1 Fig — (JPG) [file pone.0171169.s004.jpg]

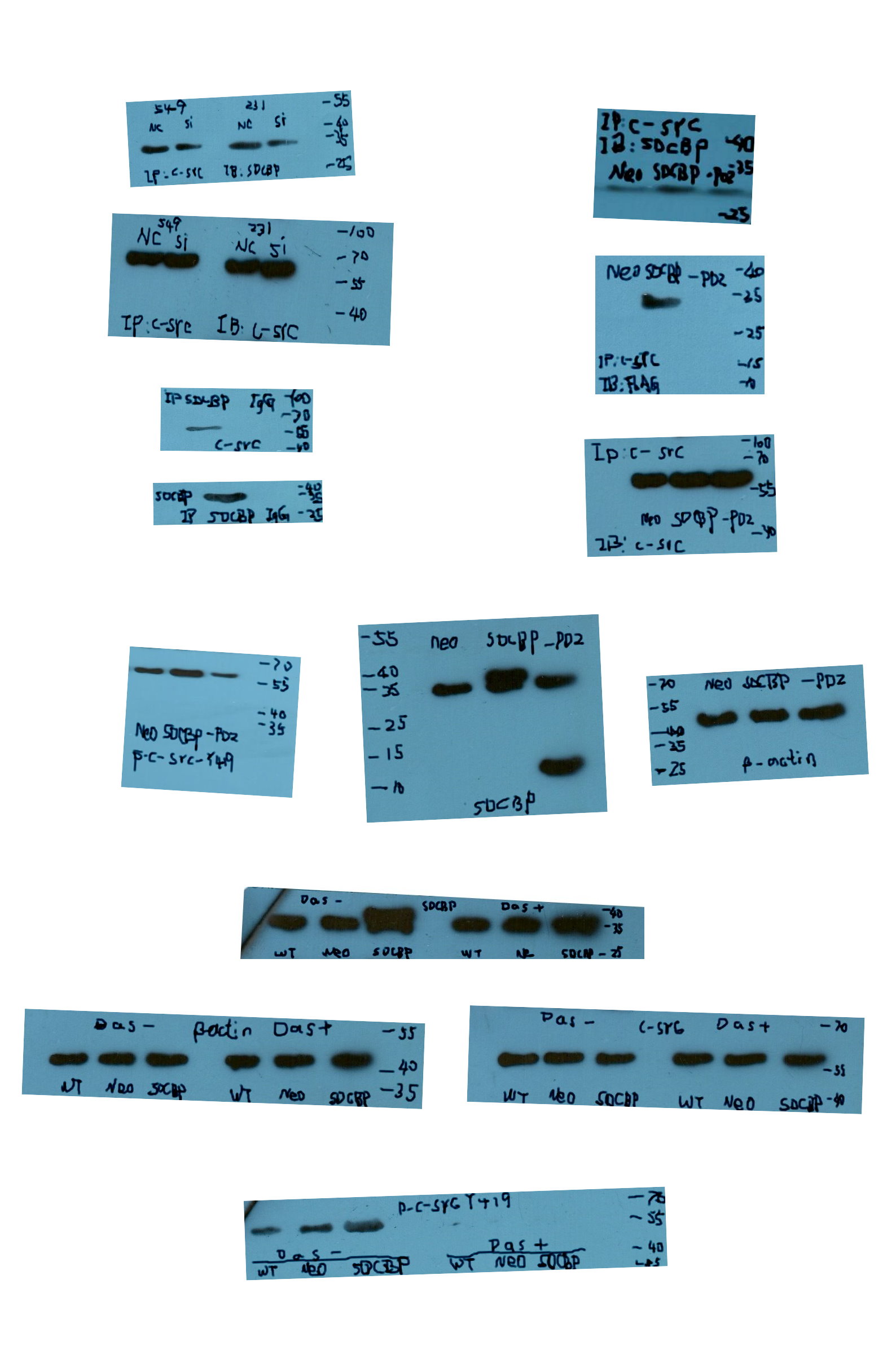

Supplement: S2 Fig — (JPG) [file pone.0171169.s005.jpg]

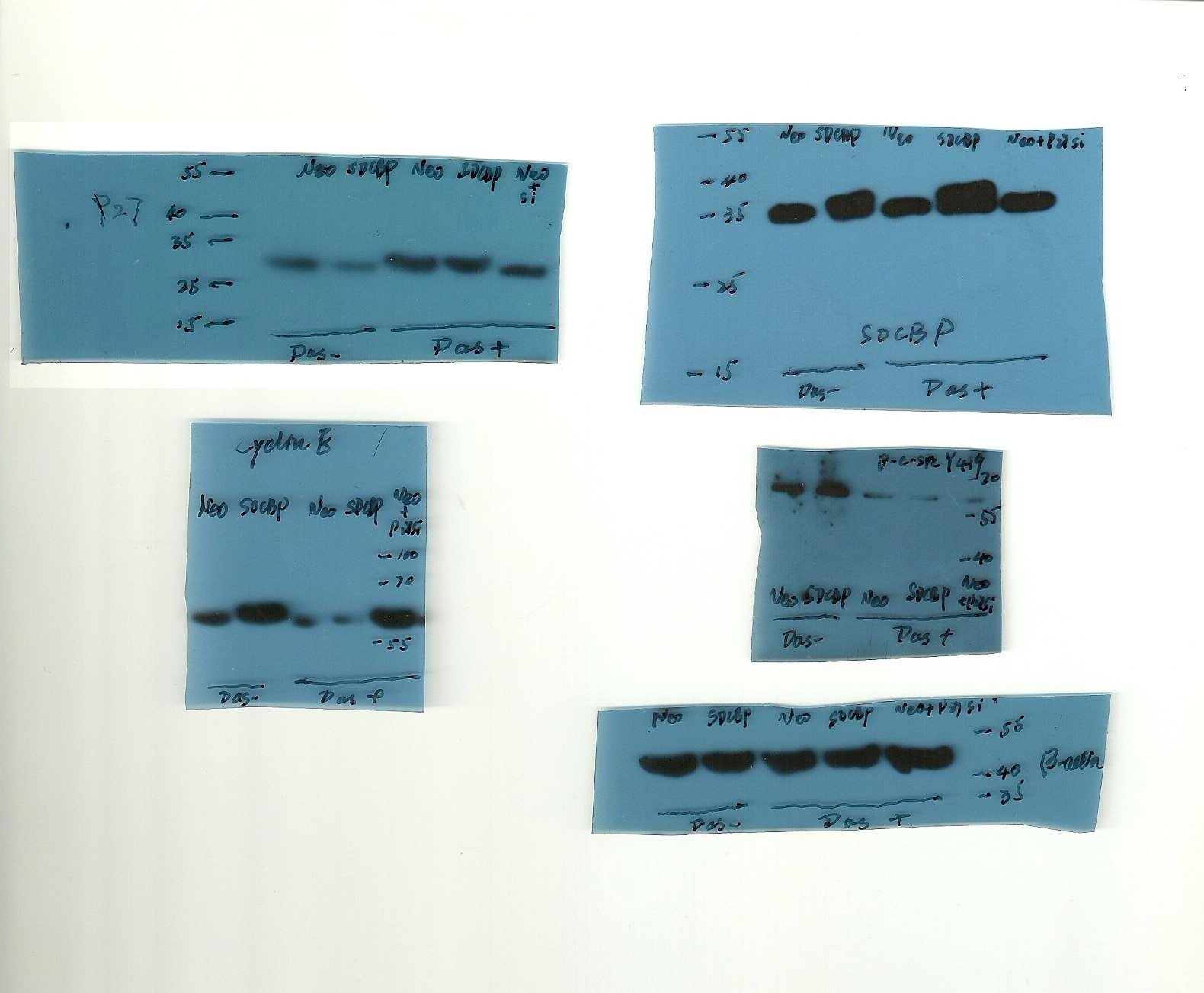

Supplement: S3 Fig — (JPG) [file pone.0171169.s006.jpg]
